# Improving access to essential health care services: the case of Israel

**DOI:** 10.1186/s13584-016-0063-x

**Published:** 2016-03-04

**Authors:** Wynand P.M.M. van de Ven

**Affiliations:** Erasmus University Rotterdam, PO Box 1738, 3000 DR Rotterdam, The Netherlands

**Keywords:** Access, Essential healthcare services, Medical loss ratio, Regulation, Private health insurance

## Abstract

In a recent article in this journal Simon-Tuval, Horev and Kaplan argue that in order to improve the protection of consumers there might be a need to impose a threshold on the medical loss ratio (MLR) for voluntary health insurance (VHI) in Israel [1]. Their argument is that VHI in Israel covers several essential services that are not covered by the mandatory benefits package due to budget constraints, while there are market failures in the VHI market that justify regulation to assure consumer protection such as high accessibility to high quality coverage.

In this commentary it will be argued that in addition to market failures there are also government failures. It is doubtful whether imposing a threshold on MLR is effective because of government failures. It can be even counter-productive. Therefore, alternative regulatory measures are discussed to promote the protection of the beneficiaries.

If essential services covered by VHI are unaffordable for some low-income people, government can extend the current mandatory basic health insurance so that it covers all essential services. If there is a budget restriction, the amount of government funds could be increased, or the health plans could be allowed to request an additional flat rate premium, set by them and to be paid by the consumer directly to their health plan. Also, effective out-of-pocket payments could be introduced. Subsidies could be given to low-income people to compensate for their additional expenses under the mandatory health insurance. If these changes are adopted, then the government would no longer be held responsible for access to benefits outside the mandatory health insurance. Accordingly, all VHI could be sold on the normal free insurance market, just as other types of indemnity insurance.

In addition, the Israeli health insurance and healthcare markets could be made more competitive by introducing procompetitive regulation. This would increase the efficiency and affordability of healthcare.

## Background

Simon-Tuval, Horev and Kaplan (SHK) argue that in order to improve the protection of consumers there might be a need to impose a threshold on the medical loss ratio (MLR) for voluntary health insurance (VHI) in Israel [1]. Their argument is that VHI in Israel covers several essential services that are not covered by the mandatory benefits package due to budget constraints, while there are market failures in the VHI market that justify regulation to assure consumer protection (which includes ensuring broad accessibility to high quality coverage). They present empirical findings about the MLR of several types of VHI in Israel and conclude that "consistent low levels of MLR are observed". Therefore, SHK argue, there might be a need to increase the extent of regulation of VHI, in particular by imposing a minimum MLR threshold.

SHK rightly conclude that regulation is necessary in order to assure access to essential healthcare services, but they incorrectly conclude that a threshold on the medical loss ratio is the right regulation. In this paper I will first argue that in addition to market failures there are also government failures and that it is doubtful whether imposing a threshold on MLR is effective because of government failures. Second, I will discuss alternative regulatory measures to improve access to essential healthcare services.

### What is the problem to be solved?

SHK mention the following characteristics and market failures of private health insurance markets that (may) necessitate the implementation of effective regulation: the desire to protect beneficiaries (such as ensuring high accessibility to quality coverage), information deficiencies, limited competition, moral hazard, adverse selection, low MLRs, concerns about insurers’ solvency, and a negative influence of private health insurance on the publicly financed health sector (e.g. unequal competition for human resources, and inefficient duplication of the services). Although it is clear from the ‘Tinbergen rule’ [2] that each policy objective should be addressed by a single tool (or, alternatively, for each policy objective there should be at least one tool), it is not clear which specific policy objective SHK aim to achieve by implementing a MLR. I assume their objective is to achieve access, for a fair price, to the essential services that are currently not covered by the mandatory benefits package due to budget constraints^1^.

### Potential government failures

Although implementing a threshold on MLR seems an easy type of regulation, the regulator can easily make mistakes in the implementation, which can make a threshold on MLR ineffective or even counterproductive.

A major problem is that it is hard to interpret an MLR. Is a low level of MLR an indicator of efficiency (low medical expenses due to good managed care) or inefficiency (high administrative expenses and waste)? [3] Robinson argues that valid interpretation of MLR is very difficult because the value of the MLR depends on several characteristics, such as the relationship between the health plan and the providers (vertical structure), the range of networks and utilization management systems it offers (product diversification), the range of buyers (individual or group insurance) to which it markets its services (channel diversification) and its geographic scope [3]. In addition SHK indicate that the MLR may strongly depend on the type of services (low layer or high layer of VHI), the number of years the product is already sold on the market (because of waiting times before reimbursement), the presence or absence of a guaranteed renewal of the contract and the size of the insurer [2]. Furthermore there are different, often inconsistent and sometimes arbitrary accounting rules used to calculate the MLR [3].

Another problem is the effect of a threshold on MLR on the insurers’ behavior. Karaca-Mandic et al. argue that insurers’ reactions may involve both strategic and unintended responses [4]. Insurers may have incentives to re-label some of their administrative expenses as quality improvement expenses (‘medical expenses’) which increases their MLR. The MLR regulation may also result in reduced cost containment efforts because insures may have less incentive to pursue efficient utilization management or to negotiate as hard on provider reimbursement after premiums are set [4].

Given such a complex relationship between the characteristics of an insurance product and the MLR, the question is whether the regulator has sufficient information, knowledge and expertise to set an adequate level of MLR. One can easily imagine that there is a substantial probability that the regulator sets the threshold inadequately. If the regulator sets the threshold too low, it is ineffective. But if the regulator sets the threshold too high, the consequences can be dramatic. In the worst case scenario insurers can go bankrupt due to the penalty they have to pay or because they do not dare to increase their premium. Other consequences can be a reduction of the benefits, quality skimping, risk selection (if that is an efficient way of reducing medical expenses while keeping the premium equal) and unfair competition between insurers with heterogeneous characteristics.

In short, in addition to the existence of market failures the implementation of a threshold for the MLR may result in serious government failures with adverse effects.

### Alternative solutions and forms of regulation

This raises the question: are there better and more effective solutions to the problem? To achieve affordability of the essential services that are currently not covered by the mandatory benefits package due to budget constraints (the policy goal), one can think of the following two alternative potential solutions: (1) extend the basic benefits package and (2) improve efficiency and affordability in the market for basic health insurance by procompetitive regulation to make this market more competitive.

First, the mandatory benefits package can be extended with all essential benefits. Consequently all VHI can be sold on the normal free insurance market, just as other types of indemnity insurance (such as car insurance and theft insurance). This is how the health insurance markets in the Netherlands are regulated. The view of the Dutch government is that because all essential benefits (i.e. benefits that are necessary, effective, cost-effective and cannot be left to the individual responsibility [5]) are included in the mandatory basic health insurance, government can no longer be held responsible for access to benefits outside the mandatory health insurance. Budget constraints for the mandatory benefits package can be solved by (1) increasing the amount of government funds available to finance the basic benefits package, either by increasing the earmarked health tax or by increasing funding out of general revenues (e.g. by increasing the annual allocation for adding new technologies to the benefits package); (2) introducing a flat rated premium set by each plan and to be paid directly by the insured to the chosen health plan (which increases premium competition among the health plans in Israel); and (3) expanding cost-sharing (deductibles, copayments, coinsurance), which both reduces moral hazard (and thereby reduces medical expenses) and shifts a part of the public expenses to private expenses. Children could be exempted from these measures. Low-income households can be subsidized to compensate for their additional expenses under the mandatory health insurance. The total public payments to the health plans plus these subsidies could be determined by the public budget constraints.

Second, efficiency and affordability on the market for basic health insurance can be improved by introducing procompetitive regulation. Although the Israeli healthcare system is often characterized as an example of the ‘managed competition model’, a comparative analysis of countries with competitive healthcare markets showed that several essential preconditions for efficiency and affordability are not (yet) fulfilled in the Israeli healthcare system [6]. In particular, Israel could improve the contestability of the market for basic health insurance and the hospital markets. Currently it is impossible or very hard to establish a new health plan in Israel or to set up a new hospital. The well-known phrase ‘Four are few; six are many’ is very applicable to the Israeli health plan market [7]. In addition there is no competition regulation that forbids anticompetitive behavior and cartels in the healthcare sector (both health plans and providers of healthcare). The competiveness of the provider markets can be substantially improved by the publication of transparent and easily understandable information on the quality of the care (and Israel has indeed begun to take steps in this direction). Inefficient risk selection by health plans can be substantially reduced by strongly improving the risk equalization system in the health plan market (which is currently primitive, from an international perspective). The implementation of a flat rate premium to be set by each health plan increases price competition among the health plans. Another advantage of allowing flat rate premiums is that health plans that provide above average quality of healthcare and therefore have above average expenses, will not go bankrupt, but can request a higher premium. In conjunction with these requests, the regulator could require the insurers to provide public information on their MLR and profits. In addition they can explain why their MLR is high or low and how they allocate their profits (premium rebates, solvency, extra benefits, or better quality of healthcare, etc.). And finally the consumer could be given more choice options of mandatory health insurance products and more ease of switching. All these measures can improve efficiency and affordability in healthcare by reducing health expenses while maintaining the quality of care, and thereby making it easier to meet the public budget constraints for the mandatory basic health insurance.

## Conclusion

Simon-Tuval, Horev and Kaplan (SHK) [1] incorrectly conclude that a threshold on the medical loss ratio (MLR) is the right regulation to assure consumer protection such as broad accessibility to high quality coverage. It is doubtful whether imposing a threshold on MLR would be effective because of government failures that may occur.

If essential services covered by VHI are unaffordable for some low-income people, government can extend the current mandatory basic health insurance so that it covers all essential services. If there is a budget restriction, the amount of government funds could be increased, or the health plans could be allowed to request an additional flat rate premium, set by them and to be paid by the consumer directly to their health plan. Also effective out-of-pocket payments could be introduced. Subsidies could be given to low-income people to compensate for their additional expenses under the mandatory health insurance. If these conditions are met, then government can no longer be held responsible for access to benefits outside the mandatory health insurance. Accordingly, all VHI can be sold on the normal free insurance market, just as other types of indemnity insurance. Because regulation of a competitive health insurance market is very complex, it is better to have one type of good and effective regulation for all essential benefits, rather than two types of different and potentially ineffective regulations (one for mandatory basic health insurance, and one apart for VHI).

In addition, the Israeli health insurance and healthcare markets could be made more competitive by introducing procompetitive regulation. This would increase the efficiency and affordability of healthcare.

## Commentary on

Simon-Tuval T, Horev T, Kaplan G. Medical loss ratio as a potential regulatory tool in the Israeli healthcare system. Israel Journal of Health Policy Research 2015;4:21

## Footnotes

1 Consequently other objectives should be achieved by other types of regulation, such as e.g. enforcing the provision of transparent information (to reduce information deficiencies), antitrust policy and making the market contestable (to make the market more competitive), cost-sharing (to reduce moral hazard), allowing risk-rated premiums (to reduce adverse selection), solvency requirements (to prevent insolvency), and improving the quality of care in the public sector (to prevent negative effects of private health insurance on the public sector).
